# How to approach clinically discordant FT4 results when changing testing platforms: real-world evidence

**DOI:** 10.1007/s12020-022-03098-5

**Published:** 2022-06-11

**Authors:** Luca Giovanella, Leonidas Duntas, Federica D’Aurizio, Hedwig Kurka, Tatjana Ammer, Christopher M. Rank, W. Edward Visser, Sjoerd A. A. van den Berg

**Affiliations:** 1 Clinic for Nuclear Medicine and Competence Centre for Thyroid Diseases, Ente Ospedaliero Cantonale, Bellinzona, and University Hospital and University of Zurich, Zurich, Switzerland; 2 Evgenideion Hospital, Unit of Endocrinology, Diabetes and Metabolism, University of Athens, Athens, Greece; 3 Institute of Clinical Pathology, Department of Laboratory Medicine, Santa Maria della Misericordia University Hospital, Udine, Italy; 4 Roche Diagnostics GmbH, Penzberg, Germany; 5 Chair of Medical Informatics, Friedrich-Alexander-University Erlangen-Nuremberg, Erlangen, Germany; 6 Academic Center for Thyroid Diseases, Department of Internal Medicine, Erasmus Medical Center, Rotterdam, Netherlands; 7 Department of Clinical Chemistry, Erasmus Medical Center, Rotterdam, Netherlands

**Keywords:** Free thyroxine, Thyroid-stimulating hormone, Thyroid dysfunction, Verification, Reference intervals, Diagnostic strategies

## Abstract

**Purpose:**

Measurement of thyroid-stimulating hormone (TSH) and free thyroxine (FT4) is important for assessing thyroid dysfunction. After changing assay manufacturer, high FT4 versus TSH levels were reported at Ente Ospedaliero Cantonale (EOC; Bellinzona, Switzerland).

**Methods:**

Exploratory analysis used existing TSH and FT4 measurements taken at EOC during routine clinical practice (February 2018–April 2020) using Elecsys® TSH and Elecsys FT4 III immunoassays on cobas® 6000 and cobas 8000 analyzers (Roche Diagnostics). Reference intervals (RIs) were estimated using both direct and indirect (refineR algorithm) methods.

**Results:**

In samples with normal TSH levels, 90.9% of FT4 measurements were within the normal range provided by Roche (12–22 pmol/L). For FT4 measurements, confidence intervals (CIs) for the lower end of the RI obtained using direct and indirect methods were lower than estimated values in the method sheet; the estimated value of the upper end of the RI (UEoRI) in the method sheet was within the CI for the UEoRI using the direct method but not the indirect method. CIs for the direct and indirect methods overlapped at both ends of the RI. The most common cause of increased FT4 with normal TSH was identified in a subset of patients as use of thyroxine therapy (72.6%).

**Conclusions:**

It is important to verify RIs for FT4 in the laboratory population when changing testing platforms; indirect methods may constitute a convenient tool for this. Applying specific RIs for selected subpopulations should be considered to avoid misinterpretations and inappropriate clinical actions.

## Introduction

The laboratory assessment of thyroid dysfunction relies on the measurement of circulating concentrations of thyroid-stimulating hormone (TSH) and free thyroxine (FT4), while free triiodothyronine (FT3) should only be required in selected cases with normal FT4 and suppressed TSH values [1]. As TSH and FT4 have a complex, non-linear relationship, small variations in FT4 may result in comparatively large variations in TSH [1, 2]. Despite some rare exceptions (e.g., central hypothyroidism, resistance to thyroid hormones, TSH-secreting pituitary adenoma, treated hyperthyroidism, and non-thyroidal illness), TSH measurement is a sensitive screening test for thyroid dysfunction and is endorsed as the best first-line strategy for detecting thyroid dysfunction in most clinical settings [3, 4].

Following abnormal TSH measurement, FT4 quantification should be added to existing laboratory requests, either automatically or based on algorithms (i.e., reflex testing) [1], reducing the number of cases for additional testing without compromising the detection of overt thyroid dysfunction. However, FT4 results can vary significantly between different assays, and though progress has been made towards the standardization of FT4 testing, technical and logistical challenges persist [1, 3–7]. Therefore, when introducing a new assay, laboratories and clinicians should work closely together to identify possible abnormalities in results.

Accordingly, the provided FT4 reference intervals (RIs) differ between manufacturers, thus a change in FT4 assay requires careful verification before the introduction of the new assay in clinical practice. There are two methods that can be used to estimate the RIs: 1) the direct method, which utilizes a cohort of healthy individuals from a reference population, and 2) the indirect method, which uses existing data from routine measurement comprising a mixed population of samples with abnormal and normal test results [8, 9]. Various influences on FT4 measurements, such as factors related to patients and medications, should be considered. Specifically, TSH and FT4 immunoassays are vulnerable to essential interferences (e.g., macro-TSH, biotin, anti-streptavidin antibodies, anti-ruthenium antibodies, thyroid hormone autoantibodies, and heterophilic antibodies) that were recently described in a systematic review in which an algorithm identifying the interferences was proposed [10]. On the other hand, the International Federation of Clinical Chemistry and Laboratory Medicine Committee for Standardization of Thyroid Function Tests (IFCC C-STFT) established reference systems for TSH harmonization and FT4 standardization and is now working with national partners on implementing these systems [11].

The Department of Laboratory Medicine at Ente Ospedaliero Cantonale (EOC; Bellinzona, Switzerland) changed thyroid function testing to Elecsys® TSH and Elecsys FT4 III immunoassays on cobas® 6000 and cobas 8000 analyzers (Roche Diagnostics International Ltd, Rotkreuz, Switzerland) in February 2018 and the RIs provided by the manufacturer were applied. Subsequently, some clinicians reported inappropriately high serum FT4 concentrations compared with corresponding TSH values. A similar phenomenon was observed at Erasmus Medical Center (Rotterdam, Netherlands) when changing thyroid function testing to the Lumipulse G1200 platform (Fujirebio Inc., Tokyo, Japan), where a comparison study against the reference measurement procedure developed by de Grande et al. [5] was undertaken (analyses not shown). Therefore, we aimed to address the practical challenges associated with changing assay and analyzer manufacturers for TSH and FT4 tests and, in particular, the verification of RIs using direct versus indirect estimation methods. An extensive analysis was performed by laboratory specialists, clinical thyroidologists, and the manufacturer (Roche). The analysis used a recently developed algorithm, refineR, which is an indirect method estimating the RI for FT4 from real-world data [8].

## Materials and methods

This was an exploratory analysis using existing TSH and FT4 measurements obtained during routine clinical practice from patients referred to EOC between February 2018 and April 2020. Anonymized data were extracted from the laboratory information system and electronic clinical files. In addition, a group of patients with complete demographic and clinical data available (including final diagnosis, current medications, and thyroid examination results [clinical and/or ultrasonographic]) were used to analyze the cause of discrepant results. Additional information (e.g., in vitro screening for conditions that could interfere with TSH and FT4 immunoassays) was also recorded. Ethical approval was provided by the EOC Scientific Advisory Board and the Tessin Ethical Committee; written informed consent from patients was waived for this study due to its retrospective design.

### Analyzers/assays

TSH and FT4 were quantified using the Elecsys TSH and FT4 III immunoassays on the cobas 6000 and cobas 8000 analyzers; both the Elecsys TSH and FT4 III immunoassays are designed for use with serum and plasma samples [12, 13]. Assay measuring ranges and RIs are summarized in Table 1.

**Table 1:** Overview of lower and higher end of the measuring range and RI for Elecsys TSH and Elecsys FT4 III immunoassays

Parameter	Limit of detection	Upper end of measuring range	Lower end of RI (2.5% quantile)	Upper end of RI (97.5% quantile)
TSH (µlU/mL)	0.005	100	0.27	4.2
FT4 (pmol/L)	0.5	100	12	22

*FT4* free thyroxine, *RI* reference interval, *TSH* thyroid-stimulating hormone

### Analysis sets

Serum sample measurements were taken from several different clinics within EOC. The age and sex of the patient, request date of the measurement, and an anonymized patient identifier were provided for each serum sample. Samples with missing measurement values were removed prior to the analysis. Individual datasets were grouped into ‘all measurements’, containing samples with both abnormal and normal TSH test results, and ‘all measurements from euthyroid patients’, containing only samples with normal TSH test results. TSH results were considered normal if all measurements were within the respective TSH RI. In all other cases, including where patients had multiple measurements both within and outside of the respective TSH RI, TSH results were considered as abnormal.

### Data analysis

Two methods for estimation of RIs were applied: direct and indirect.

#### Estimation of RIs using the direct method

RIs were estimated using the ‘all measurements from euthyroid patients’ pooled dataset, containing only samples with normal TSH test results based on the definition above. Estimation was performed for the whole group as well as for subgroups based on sex, age, and site. Each patient was analyzed only once; if several samples from one patient were sorted into a respective evaluation group, only the sample with the earliest request date was included in the calculations. For determination of RIs, sample percentiles were calculated using a rank-based quantile estimation in the statistical programming language R [14]. Two-sided distribution-free conservative confidence intervals (CIs) for percentiles were estimated using the method of Hahn and Meeker [15]. In this approach, ≥120 samples per cohort were needed to estimate the central 95% RI (2.5–97.5% quantile) and its CI with sufficient statistical confidence [16].

#### Estimation of RIs using the indirect method (refineR algorithm)

RIs were also estimated using an indirect method, the refineR algorithm described in Ammer et al. 2021 [8]. In contrast to the direct method, the indirect method used the ‘all measurements’ dataset containing samples with abnormal and normal TSH test results for the estimation of RIs; information on the pathology status of samples was not available to the algorithm, but each patient was analyzed only once. Analysis of subgroups was not applied, as the sample sizes of subgroups were not sufficient for a robust estimation with the refineR algorithm.

The refineR algorithm [8] assumes that routine data consist of results from samples with abnormal and normal test results, with the latter in the majority. It also assumes that the distribution of the samples with normal test results can be modeled with a Box–Cox transformed normal distribution, which can accommodate normal as well as skewed distributions. The Box–Cox transformed normal distribution is defined by three parameters: µ (mean value of normal distribution), σ (standard deviation of normal distribution), and λ (power parameter describing the skewness of the distribution). To find the optimal model defined by the optimal parameter set µ*, σ*, and λ*, a multi-level grid search is employed; the parameter set µ*, σ*, and λ* is considered optimal when it reveals a maximum log-likelihood to describe the histogram of the routine data in a central concentration region.

An inverse Box–Cox transformation was applied using the optimal transformation parameter λ* on the 2.5% and 97.5% quantiles of the normal distribution defined by µ* and σ*, and the desired RIs were obtained (in particular, the central 95% region of the estimated distribution of normal samples). A bootstrap-based approach was used to calculate CIs for the RIs. Drawing on bootstrap samples from the dataset (*n* = size of the dataset), the parameter optimization of µ*, σ*, and λ* was repeated 200 times. The 95% CI was obtained as the central 95% region of the 200 RIs estimated from bootstrapping.

## Results

### RI evaluation

**Fig. 1:**
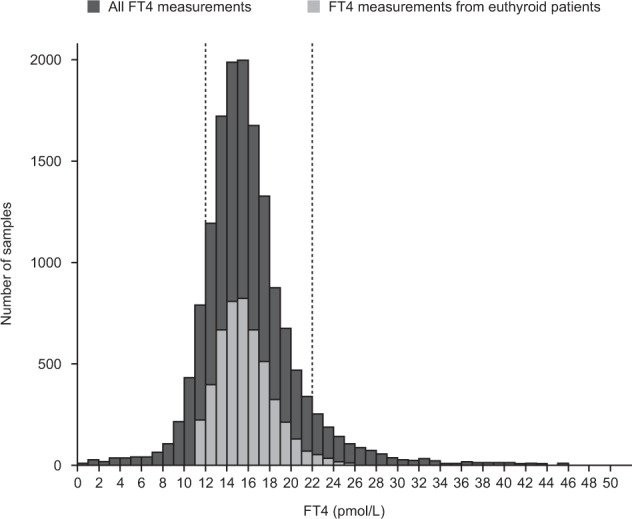
FT4 measurements in all samples (*n* = 15,213) and euthyroid patients (*n* = 5111). *FT4* free thyroxine. Vertical dotted lines at 12 and 22 pmol/L FT4 show the normal range provided by the manufacturer. Only one sample per patient was included for euthyroid patients

In all patients with a normal TSH value (0.27–4.2 µlU/mL; *n* = 5111), the majority of FT4 measurements were also within the normal range (12–22 pmol/L) provided by the manufacturer (90.9%, *n* = 4648; Fig. 1).

**Fig. 2:**
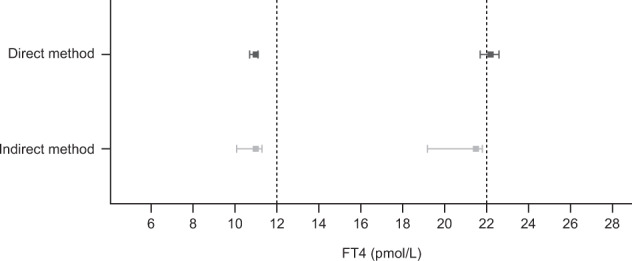
Determination of RIs for FT4 using the direct and indirect methods. *FT4* free thyroxine, *RI* reference interval. Vertical dotted lines at 12 and 22 pmol/L FT4 show the normal range provided by the manufacturer. Plots show estimated lower (2.5% quantile) and upper (97.5% quantile) RI limits with 95% confidence intervals

For FT4 measurements, the CIs for the estimated value of the lower (2.5% quantile) end the RI derived from the direct and indirect estimation methods overlapped; however, both estimates were lower than the estimated value listed in the immunoassay method sheet (Table 2, Fig. 2). For the upper (97.5% quantile) end of the RI, the CIs obtained from the direct and indirect methods overlapped by 0.1 pmol/L; the CI obtained by the direct method encompassed the estimated value listed in the method sheet while the CI obtained by the indirect method was lower than the estimated value in the method sheet (Table 2, Fig. 2).

**Table 2:** Determination of local RIs for FT4

Method	Samples, *n*	2.5% quantile (pmol/L)				97.5% quantile (pmol/L)					
		Estimate	LCL	UCL		Estimate		LCL		UCL	
Direct	5111	11	10.7		11.1		22.2		21.7		22.6
Indirect (refineR)	9065	11	10.1		11.3		21.5		19.2		21.8
Method sheet	801	12	NA		NA		22		NA		NA

*FT4* free thyroxine, *LCL* lower confidence limit, *NA* not available, *RI* reference interval, *UCL* upper confidence limit

### Analysis of divergent results above the manufacturer upper RI

Out of 9065 patients, 306 patients with complete demographic and clinical data available showed high (>22 pmol/L) levels of FT4 with normal TSH levels; the causes of discrepant results were identified in 263 of these 306 patients (Table 3). The most common reason for increased FT4 with normal TSH was use of thyroxine therapy (72.6%, *n* = 191); other reasons for the discrepancy between FT4 and TSH levels included use of amiodarone (14.4%, *n* = 38), other drugs (7.6%, *n* = 20), and analytic interferences (5.3%, *n* = 14).

**Table 3:** Causes of divergent results between high FT4 and normal TSH levels (*n* = 306)

FT4 values (pmol/L)	Cause unknown, *n*	Cause known, *n*				
		Total	Thyroxine	Amiodarone	Other drugs	Analytic interferences
22.0–23.9	16	149	132	13	1	3
24.0–25.9	11	68	47	14	5	2
26.0–27.9	6	22	8	6	5	3
28.0–29.9	7	12	4	5	1	2
30.0–31.9	3	6	0	0	3	3
32.0–46.0	0	6	0	0	5	1
Total, *n* (%)	43 (14.1)	263 (85.9)	191/263 (72.6)	38/263 (14.4)	20/263 (7.6)	14/263(5.3)

*FT4* free thyroxine, *TSH* thyroid-stimulating hormone

## Discussion

As FT4 levels determined using analyzers from different manufacturers cannot be compared, specific RIs per method are required, with a need for standardization. While method sheet RIs may be used, it is important that laboratories verify the RIs at a local level. Therefore, if a laboratory observes unexpected results, the RIs should be assessed and appropriate criteria should be discussed, implemented, and periodically updated. When RIs are updated, context and education should be provided by clinical chemists for all clinicians to avoid overdiagnosis of thyroid dysfunction [17].

In this study, while some inappropriately high serum FT4 concentrations compared with corresponding normal TSH values were seen following a change in thyroid function testing at EOC, our analyses found that the manufacturer RIs were appropriate for the laboratory population. RIs were calculated from routine clinical data using two different methods, direct and indirect (refineR algorithm). Although the resulting RIs were comparable between the methods, CIs for the upper end of the RI only overlapped by 0.1 pmol/L. This observation may be due to the fact that FT4 levels can increase substantially for several hours after levothyroxine treatment intake, with minimal change in TSH [18]. Given that the indirect method utilizes large data sources that are more easily accessible and directly target the local population, it can be a valuable tool for assessing the suitability of RIs.

### Prevention of divergent results

Serum TSH and FT4 concentrations and RIs may differ depending on the assay method, and FT4 levels often show greater variability than TSH levels [19, 20], though the extent of variation has not been systematically evaluated. A previous study found that 10.3% of patients treated with levothyroxine had high FT4 concentrations alongside a normal TSH measurement [18]. In our study, we found that thyroxine therapy was the most common cause (72.6%) of increased FT4 levels when TSH levels were normal.

TSH has a broad RI and can achieve an accurate diagnosis of hypothyroidism when evaluated as a single laboratory parameter [3, 4]. However, when TSH is abnormal, additional testing is required before treatment decisions can be made [21]. Testing for FT4 is recommended if TSH levels suggest hypothyroidism, and testing both FT4 and FT3 is recommended if TSH levels suggest hyperthyroidism [21]. If there is clinical suspicion of secondary hypothyroidism or a rare disorder, it is advised to simultaneously measure TSH and FT4. In a large, unselected, community-dwelling population-based study, Schneider et al. [22] found that a two-step reflex testing approach (i.e., assessing FT4 only if TSH is outside the RI) could eliminate unnecessary FT4 testing in up to 93% of participants compared with a one-step approach. Previous studies using a similar approach to Schneider et al. also reported that unnecessary FT4 testing could be reduced by ~90–99.6% [23, 24]. The study by Schneider et al. found that most (85%) patients with normal TSH results but FT4 outside the RI (3.8% of the whole study population) were within 2 pmol/L of the upper or lower limits of the FT4 RI and could be considered likely to be healthy euthyroid outliers.

### Verification of RIs

Verification is necessary when a laboratory wishes to adopt an established RI supplied by a manufacturer or another laboratory for the same or similar analytical system. This verification involves determining reference values for at least 20 individuals judged to be representative of the adopting laboratory’s healthy population [16, 25, 26]. Guidelines [16, 27] stipulate that if, after repeated sampling, more than two (10%) reference values fall outside the established RI, it is an indication that the population served by the laboratory differs significantly from that used to set the manufacturer’s RI; in this case, a local RI should be established. Due to the small sample size (*n* = 20), the statistical uncertainty of this approach is high. Furthermore, the statistical design of the approach prevents detection of a RI that is too wide. Consequently, alternative approaches such as the indirect method are required to independently verify the RIs.

### Definition of RIs

Direct and indirect estimation methods for RIs result in similar but not equal results. Differences in the tested populations (e.g., nationality, age distribution, sex distribution, and sample size), as well as whether or not the site has other departments, can lead to different RIs (e.g., calculating RIs for the same assay in neighboring hospitals, one academic and one non-academic). In addition, each estimation technique has different strengths and limitations. When using the direct method, the applied filtering using only samples with normal TSH values is limited as a certain fraction of discordant samples with normal TSH values and discordant FT4 levels is to be expected, which can lead to a suboptimal estimation of the RIs [9]. Using the indirect method has some advantages over the direct method, including large data sources that are more easily accessible, analysis that directly targets the local population, and preanalytical and analytical factors that reflect those used in the local laboratory [28]. However, a limitation of the indirect method is that separation of abnormal and normal distributions may not be perfect (i.e., patients with untreated thyroid dysfunction and patients who have been successfully treated for thyroid dysfunction may have been included when establishing a RI to guide diagnosis and treatment), resulting in a potential bias of the estimated RI [9, 29]. Despite this, no differences were found between our general population (including patients from Nuclear Medicine and Endocrinology) and the thyroid healthy population. Additionally, in order to achieve the most robust results using the indirect method, ideally only a small proportion of the samples (<20%) should have abnormal measurements; however, it has been shown that the refineR algorithm can still achieve reliable results with a higher fraction of abnormal test results [8].

## Conclusions

When changing platforms to test thyroid function parameters, it is important to verify established RIs in the laboratory population. The indirect method (refineR algorithm) is useful to estimate new RIs from easily accessible large samples rather than filtered samples as required for the direct method; however, each method has its own strengths and limitations.

## Data Availability

The study was conducted in accordance with applicable regulations. Ethical approval was provided by the EOC Scientific Advisory Board and the Tessin Ethical Committee; written informed consent from patients was waived for this study due to its retrospective design. For more information on the study and data sharing, qualified researchers may contact the corresponding author, Prof. Dr. Luca Giovanella, MD PhD (luca.giovanella@eoc.ch).
